# Generative AI Chatbots as Digital Adjuncts for Sexual Health Information After Prostate Cancer in Men Who Have Sex With Men: Auto-Netnographic Study

**DOI:** 10.2196/81745

**Published:** 2026-02-09

**Authors:** Mats Christiansen, Henrik Eriksson, Lisbeth Fagerström

**Affiliations:** 1 Department of Public Health and Caring Science, Uppsala University, BMC, Husargatan 3, Uppsala, 752 37, Sweden, 46 184716620; 2 Department of Natural and Health Sciences, The Faculty of Science and Engineering, Åbo Akademi University, Vaasa, Finland; 3 Section for Health Promotion and Care Sciences, University West, Trollhättan, Sweden

**Keywords:** artificial intelligence, auto-netnography, generative AI chatbots, netnography, prostate cancer, sexual minority men

## Abstract

**Background:**

Sexual health concerns following prostate cancer treatment are common yet often insufficiently addressed in clinical practice, particularly among men who have sex with men. These individuals may face additional barriers stemming from heteronormative assumptions, limited disclosure, and a lack of culturally tailored information. As generative artificial intelligence (GenAI) chatbots become increasingly accessible, patients are using these systems to seek sensitive health information outside traditional care settings. While prior research has focused on the accuracy and safety of chatbot-generated health advice, less attention has been paid to how responses are framed and experienced in sexual minority contexts.

**Objective:**

This study aimed to describe and compare how 4 GenAI chatbots respond to questions about sexual health following prostate cancer treatment, with a focus on the needs of a gay man, and to interpret these responses using netnographic and actor-network theory perspectives.

**Methods:**

A qualitative exploratory study using auto-netnography was conducted. In February–March 2025, the first author interacted once with 4 widely used GenAI chatbots—ChatGPT (GPT-4o; Open AI), Claude (3.5 Sonnet; Anthropic), Copilot (GPT-4 Turbo; Microsoft), and Gemini (2.0 Flash; Google)—while assuming the role of a simulated “mock patient.” Two standardized prompts were used verbatim across all platforms: an initial prompt addressing sexual health concerns after prostate cancer treatment and a supplementary prompt focusing on sexual minority–specific issues, including same-sex practices. Chatbot outputs were treated as system-generated data and analyzed qualitatively, integrating system-generated text with reflexive experiential engagement and attention to interactional framing, emotional attunement, specificity, and performative features. The analysis did not assess clinical effectiveness, safety, or generalizability.

**Results:**

Across platforms, chatbot responses addressed treatment-related sexual health concerns using generally inclusive language, with variation in emotional tone, specificity, and cultural sensitivity. Interactional features included the scope and framing of clinical information, encouragement of dialogue, self-care advice, and explicit discussion of same-sex sexual practices. No obvious fabricated claims were identified; however, contextual inaccuracies were observed. Responses were mapped along 2 intersecting continua—logical-to-empathetic orientation and general-to-specific framing—yielding 4 interactional styles: structured overview, rational clarity, compassionate perspective, and compassionate precision. This 4-quadrant framework served as an interpretive heuristic and does not constitute an evaluation of quality or effectiveness.

**Conclusions:**

The findings indicate that contemporary GenAI chatbots, when used as digital adjuncts, may enact communication styles that can be perceived as supportive, culturally sensitive, and LGBTQI+ (lesbian, gay, bisexual, transgender, queer, and intersex)-inclusive in specific sexual health interactions. Although these systems lack ethical consciousness and cannot replace professional care, their performative responses may complement clinical practice by facilitating reflection and access to sensitive information. The study highlights how care-like meanings may emerge through sociomaterial interactions between users and artificial intelligence systems rather than demonstrating generalized performance or clinical reliability.

## Introduction

Sexual health concerns following prostate cancer treatment are common yet often insufficiently addressed in clinical practice, particularly among men who have sex with men. Sexual minority patients may face additional challenges stemming from heteronormative assumptions, limited disclosure to health care providers, and a lack of culturally tailored information and support [1-5]. As a result, men who have sex with men treated for prostate cancer may experience unmet informational and psychosocial needs related to intimacy, sexual practices, and identity-specific concerns.

Digital technologies have become increasingly prominent sources of health information, supplementing traditional clinical encounters. Alongside websites, forums, and telehealth services, generative artificial intelligence (GenAI) chatbots are now widely accessible to patients seeking health-related guidance. These conversational systems can generate responsive, personalized text and simulate dialogic engagement, making them particularly attractive for sensitive topics, such as sexual health. At the same time, concerns have been raised about the accuracy, bias, and reliability of chatbot-generated health information in both clinical and lay contexts [6-10].

Most existing research on GenAI in health contexts has focused on evaluating the correctness, safety, and technical performance of chatbot-generated outputs, often through expert benchmarking or comparison with established clinical guidelines [9,10]. While such studies are essential, they offer limited insight into how health-related responses are framed, enacted, and experienced in interaction, particularly in sensitive, identity-linked domains, such as sexual health. Less is known about how GenAI chatbots address sexuality, intimacy, and same-sex practices after prostate cancer treatment, or how interactional styles may vary across platforms when responding to identical prompts.

Qualitative and nursing-oriented research has emphasized that health communication involves not only the accuracy of information but also interactional framing, emotional attunement, and relational context [11-13]. From sociomaterial and actor–network perspectives, digital technologies can be understood as nonhuman actors that participate in enacting meanings and practices through interaction rather than merely transmitting information [14-17]. This perspective foregrounds how health-related meanings emerge relationally through specific configurations of users, technologies, and contexts.

In nursing science, particularly within the Nordic caritative caring tradition, caring is understood as an ethical and relational practice grounded in responsibility, dignity, and communion rather than solely in information provision or technical support [18,19]. This distinction offers an important reference point for interpreting artificial intelligence (AI)–mediated interactions that may resemble caring communication yet do not constitute care in a caritative sense. Building on this tradition, Andtfolk [20] has examined the possibilities and limitations of care technologies, emphasizing that digital and robotic systems may simulate aspects of caring interaction without ethical responsibility, consciousness, or the capacity for genuine caring communion.

Rather than evaluating whether GenAI chatbots provide accurate or safe medical advice, this study examines how responses from GenAI chatbots are framed, enacted, and experienced in a situated sexual health interaction with a gay man after prostate cancer treatment. By adopting an auto-netnographic approach, the study attends to both system-generated text and the researcher’s reflexive, experiential engagement with the interaction [13,21]. This approach enables exploration of interactional features, such as emotional attunement, specificity, and inclusivity without making claims about clinical effectiveness or generalizability.

The aim of this study was to describe and compare how 4 GenAI chatbots respond to questions about sexual health following prostate cancer treatment, with a focus on the needs of a gay man, and to interpret these responses using netnographic and actor–network theory perspectives.

## Methods

### Study Design

This study used an auto-netnographic qualitative design to explore situated interactions with GenAI chatbots [15,17,21]. Auto-netnography was chosen to enable reflexive engagement with chatbot responses and to examine how interactional framing, tone, and performativity are enacted in a specific health-related inquiry. The study followed the iterative netnographic process described by Kozinets, including initiation, immersion and interaction, analytic integration, and reflexive interpretation [17]. The study was not designed to evaluate clinical effectiveness, safety, or generalizable performance of chatbots, but rather to explore how responses are produced and experienced in a particular interactional context.

This study was reported in accordance with Standards for reporting qualitative research (SRQR) and the Chatbot Health Advice Reporting Tool (CHART) reporting guideline [22,23].

### Chatbot Selection and Study Context

Four widely used GenAI chatbots were included: ChatGPT (GPT-4o, free version; Open AI), Claude (Claude 3.5 Sonnet, free version; Anthropic), Copilot (GPT-4 Turbo, enterprise subscription; Microsoft), and Google Gemini (2.0 Flash, free version). These chatbots were selected for their public availability and relevance to contemporary health information-seeking. All interactions were conducted via the chatbots’ web interfaces between February and March 2025, and no technical limitations were encountered during data collection.

Interactions were carried out using a simulated “mock patient” persona enacted by the first author, drawing on his lived and professional experience as a gay man and a specialist nurse. This methodological approach was used to elicit relevant chatbot responses and does not constitute data from a real patient. Accordingly, chatbot outputs were treated as system-generated data rather than human participant data.

### Prompting and Interaction Procedure

Data collection began with a standardized initial prompt presented verbatim to all 4 chatbots (Textbox 1). The prompt described a gay man in his early fifties, recently diagnosed with prostate cancer, seeking information on how different treatment options might affect sexual health, including concerns about erections, orgasm, intimacy, and sex with a male partner.

Textbox 1. **Standardized initial prompt.**

I’m a 53-year-old gay man, and I’ve been diagnosed with prostate cancer. Will be treated with either radiotherapy or surgery, both would likely include hormonal therapy as well. I’m concerned about my sex life after treatment. Can you help me think this through?

Following the initial prompt, each chatbot generated a response and, in some cases, asked brief clarification questions (eg, about specific concerns or preferences). Follow-up replies were limited to brief clarifications (eg, one-sentence responses) and did not introduce new topics beyond the prompts. These follow-up exchanges were neither standardized nor the primary focus of the analysis.

After the initial interaction, a second standardized supplementary prompt was presented verbatim across all platforms (Textbox 2). This prompt explicitly addressed sexual minority–specific considerations, including anal sex, insertive and receptive roles, partner communication, and whether health care providers typically address these topics.

Textbox 2. **Standardized supplementary prompt**

Are there any specific things I need to consider as a gay man regarding my sex life after treatment for prostate cancer?

The standardized prompts used across all 4 chatbots are presented verbatim.

No modifications were made to the wording of either prompt across platforms. All interactions occurred in a single session per chatbot. Chatbot responses to both standardized prompts were captured verbatim and transferred to a spreadsheet for analysis. No responses were edited, filtered, or corrected prior to analysis. The analytic focus was on how responses were framed, enacted, and performed in this specific interactional context, rather than on response stability, reproducibility, or clinical correctness.

### Positionality and Reflexivity

The first author identifies as a gay man and is a specialist nurse with longstanding clinical and research experience in sexual health and prostate cancer. The coauthors served as project supervisors; HE has expertise in netnographic and qualitative research, and LF has expertise in analytic and interpretive methods. This positionality informed the selection of prompts and the analytic focus. Reflexive field notes were recorded during and after interactions, capturing immediate impressions and evolving interpretations. Preliminary analyses were discussed among all authors to support reflexivity and analytic transparency.

### Analytic Approach

The analysis was qualitative and interpretive. Consistent with auto-netnographic methodology, the first author’s experiential engagement with the interaction was integral to the analytic material [21]. Rather than using a formal, predefined thematic coding framework or quantification, the analysis focused on how responses from GenAI chatbots were framed, how emotional attunement and specificity were enacted, and how interactional styles varied across platforms. The analysis was informed by netnographic principles and actor–network theory, emphasizing enactment and performativity in human–technology interaction [14-17]. Auto-netnography was used to capture the lived, situated experience of interacting with GenAI chatbots, including affective responses, interpretive judgments, and reflexive meaning-making that extend beyond the textual content of chatbot outputs [15,21].

While the chatbot responses constitute system-generated text, the analytic material also included the researcher’s experiential engagement with the interaction, which informed the interpretation of tone, responsiveness, and perceived relevance in context. The analysis began with a naïve reading of all prompt responses to gain an immediate overall understanding of the material. In the next step, responses were placed side by side to identify similarities and differences. As part of this comparative phase, a sentence-level content analysis was performed through line-by-line coding, enabling a more granular examination of interactional features and thematic patterns.

Initial line-by-line coding focused on interactional features, including tone, emotional attunement, specificity, and forms of guidance. These features were compared across chatbot responses to identify recurring contrasts, which were abstracted into 2 intersecting dimensions, namely empathetic–rational orientation and general–specific framing. The 4-quadrant analytic map was then developed as a heuristic to visualize and interpret how different interactional styles were enacted across platforms.

**Figure 1.**
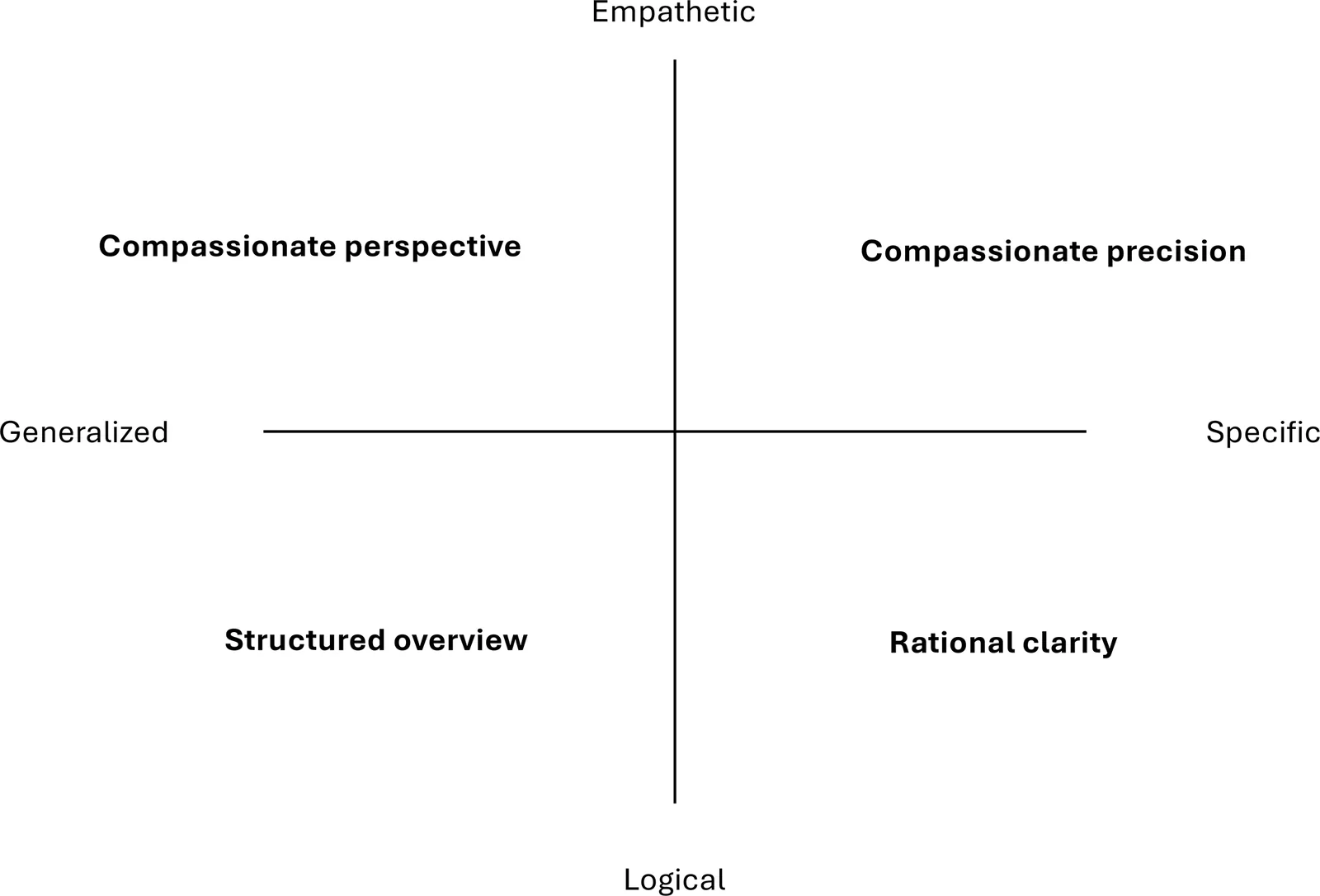
An interpretive heuristic for describing chatbot interactional styles.

During the comparative phase, the theoretical framework and the first author’s preunderstanding were mobilized to pose reflexive questions about the prompt responses, consistent with contemporary netnographic and auto-netnographic approaches to AI-mediated interaction [15,21]. Two questions guided the analysis: (1) What does this mean from a theoretical lens? and (2) How can I understand this (as a gay man)? These questions served as reflexive lenses for heuristic interpretation across both disciplinary and experiential horizons. This process revealed substantial variation in how the prompts addressed their intended recipients, in terms of both language and tone. To organize these comparative interpretations, the 4-quadrant analytic map (Figure 1) was used as an interpretive lens to present the study’s findings, rather than as a classificatory or evaluative model.

Assessments of accuracy, inclusivity, and empathy were pragmatic and reflexive. Accuracy was defined pragmatically as the absence of obvious factual errors and general alignment with established clinical knowledge familiar to the authors, rather than as a formally validated assessment. No formal scoring instruments, interrater reliability testing, or expert panel validation were used. Inclusivity was pragmatically assessed by using nonheteronormative assumptions, gender-neutral partner language, and explicit acknowledgment of same-sex practices when prompted.

### Ethical Considerations

The study did not involve human participants. Chatbot outputs were treated as system-generated data. The use of a mock patient persona was a methodological strategy to elicit relevant responses, not a form of deception involving human participants. Ethical approval was obtained from the Swedish Ethical Review Authority (Dnr 2024-02924-02).

## Results

The 4 GenAI-powered chatbots responded to the prompts with text addressing the specific areas outlined in the prompts (Multimedia Appendix 1). All chatbots’ GenAI responses included follow-up questions or invitations to continue the dialogue. The findings include the following themes: (1) the content of the chatbot replies, (2) expressions of empathy in chatbot responses, (3) encouraging dialogue, (4) providing self-care advice, (5) discussing same-sex sexual practices, and (6) tonality and cultural sensitivity.

### Content of the Chatbot Replies

Across all platforms, chatbot responses addressed treatment-related sexual health concerns using inclusive language, with variation in emotional tone and specificity. Gemini’s response cited 4 published scientific papers, all of which are available with open access. Across platforms, the responses addressed commonly reported treatment-related sexual health concerns. No prominent examples of hallucinated content were identified in the analyzed outputs. However, some errors were present, such as links to support groups that mistakenly listed Swedish Hospital Services, a health service in the United States, rather than Sweden. Although occasional caveats noted that the support groups were based in the United States or Australia, it was also mentioned that some could offer online support conversations.

Building on broader discussions linking sexual health to prostate cancer treatments, a second prompt was added to examine the specific sexual issues faced by a gay man undergoing treatment (refer to Multimedia Appendix 2).

*It’s important to acknowledge that while the core medical aspects of prostate cancer treatment are the same for all men, there are specific considerations for gay men regarding their sexual health. Here’s a breakdown of those key points:...*

[Gemini]

### Expressions of Empathy in Chatbot Responses

After the initial prompt, all chatbots responded in ways that conveyed empathy, compassion, and concern through language and tone, and an appreciation for the user’s sharing of personal information. The responses used language that conveyed empathy and engagement:

*I appreciate you sharing this with me. You’re facing a big decision, and it’s completely understandable to be concerned about how treatment might affect your sex life*.

[ChatGPT]

*I’m really sorry to hear about your diagnosis. It’s completely understandable to be concerned about how treatment might affect your sex life. Let’s break down some of the potential impacts and ways to manage them*.

[Copilot]

Both of these excerpts included language commonly associated with empathic communication. However, some responses may appear more detached, emphasizing facts:

*It’s completely understandable that you’re concerned about how prostate cancer treatment might affect your sex life. This is a common and valid concern for many men facing this diagnosis. Here’s a breakdown of factors to consider, and how to approach this:…*

[Gemini]

### Encouraging Dialogue

The chatbots promoted interaction by asking open-ended questions to better engage the user and gather more detailed responses for consideration:

*Would it be helpful to discuss specific strategies for maintaining intimacy during periods when hormone therapy might affect your libido or sexual function? Many men find creative ways to stay sexually active and maintain connections with partners even when dealing with these challenges*.

[Claude]

*Would you like to talk through the potential effects of each treatment in more detail? Or are you more interested in strategies for maintaining a fulfilling sex life post-treatment?*

[ChatGPT]

In these responses, follow-up questions expanded the scope of topics addressed.

### Providing Self-Care Advice

There was also advice on self-care in the responses. Several of the chatbots suggested pelvic floor exercises to improve orgasm quality:

*Enhancing Orgasms:*

*Kegel Exercises: Strengthening pelvic floor muscles may help improve orgasm intensity and control leakage.*

*Mindful Arousal Techniques: Engaging in sensual touch, breathwork, and fantasy can enhance pleasure.*

*Lubrication: Can help with sensation changes and dryness.*

[ChatGPT]

### Discussing Same-Sex Sexual Practices

Regarding same-sex sexual practices (Multimedia Appendix 2), all chatbots provided explicit descriptions of potential changes related to these practices.

*Anal Sex:*

*If you engage in receptive anal sex, this can generally be resumed after healing from either surgery or radiation (usually 4‐6 wk for surgery, timing varies for radiation).*

*If you engage in insertive anal sex, erectile challenges may affect this more significantly than some other forms of sexual activity.*

*Some men find they need to adjust their preferred sexual roles based on treatment effects*.

[Claude]

### Tonality and Cultural Sensitivity

However, the chatbots varied in tone and vocabulary. Gemini and Copilot maintained a professional, somewhat distant tone, while ChatGPT adopted a friendlier, more collegial style. The language reflected nonheteronormative sexual practices; for example, ChatGPT mentioned “Navigating hookup culture,” which allows responses sensitive to different cultural backgrounds and recognizes that not everyone is in a committed or monogamous relationship.

Claude and Copilot asked about current partners to encourage communication and support:

*Also, do you have a partner who’s part of these discussions? Partners can be valuable allies in recovery and adaptation*

[Claude]

*Openly discuss any changes in your sexual function or desires with your partner. This can help maintain intimacy and understanding in your relationship*

[Copilot]

All the chatbots consistently used gender-neutral, inclusive language.

### Care-Related Language Interpreted Through a 4-Quadrant Analytic Map

The enactment and performativity of GenAI can be mapped along two continua: (1) one from general to specific information and (2) the other from logical to empathetic responses. The 4-quadrant framework should be understood as an interpretive heuristic rather than a validated typology. It was derived from a comparative reading of chatbot responses and is intended to support interpretation of how interactional styles may vary along two intersecting dimensions, including degree of emotional attunement (logical to empathetic) and degree of specificity (general to tailored).

The interactor was prompted to provide additional information to give the chatbot broader context for engagement. In this case, the chatbot resembled an information trader, which is at the logical end of the spectrum. The compassionate aspect of GenAI places it closer to the empathetic end.

To illustrate how interactional variation was interpreted in the analysis, four analytically derived positions are described below.

1. Structured overview: Responses are characterized by logically organized general information about treatments and side effects, with limited emotional engagement or personalization.
2. Compassionate perspective: Responses acknowledge emotional concerns and the broader life context while remaining relatively general in scope.
3. Compassionate precision: Responses combine emotional attunement with tailored, context-sensitive guidance, including explicit references to same-sex practices or minority stressors.
4. Rational clarity: Responses emphasize factual explanations, references, and clinical reasoning, with minimal affective language (Figure 1).

Three of the GenAI chatbots began with compassionate responses, expressing concern and saying they were “sorry” to hear of the diagnosis. The fourth adopted a more distant or professional tone:

*It’s completely understandable that you’re concerned about how prostate cancer treatment might affect your sex life. This is a common and valid concern for many men facing this diagnosis. Here’s a breakdown of factors to consider, and how to approach this*.

[Gemini]

In the excerpt above, the chatbot acknowledges the interactor’s concern but shows less empathy. This approach is more logical and provides a structured overview, yet it is more general and does not address the interactor’s specific needs.

The chatbots played a performative role in engaging with and questioning the interactor, prompting them to provide additional details. They offered information while also seeking clarification and any further necessary data. Here, the interactional pattern resembles descriptions of person-centered communication, demonstrating compassionate precision, for example, when the chatbots used inclusive language and suggested practices for different sexual positions.

*Yes, there are several important considerations specific to gay men dealing with prostate cancer treatment:*

*Anal Sex:*

*If you engage in receptive anal sex, this can generally be resumed after healing from either surgery or radiation (usually 4‐6 wk for surgery, timing varies for radiation)*

*If you engage in insertive anal sex, erectile challenges may affect this more significantly than some other forms of sexual activity*

*Some men find they need to adjust their preferred sexual roles based on treatment effects*.

[Claude]

*Your sex life will likely change, but that doesn’t mean it has to end. Many men discover new ways to experience pleasure, whether through adapting physically, redefining intimacy, or exploring different forms of touch and connection. If you’d like, I can help you find LGBTQ+-friendly doctors, sex therapists, or resources specific to your area*.

[ChatGPT]

Some of the chatbots attempted to provide evidence-based responses by citing open-access scientific journal papers. This approach was less compassionate and focused on presenting facts, references, and links from external sources. This illustrates rational clarity.

*Yes, there are some specific considerations for gay men regarding their sex life after prostate cancer treatment. Here are a few key points:*

*Communication with Healthcare Providers*

[Copilot]

This compassionate perspective offered encouragement and suggestions for addressing these issues, such as:

*Specific Considerations for Gay MenResearch indicates that gay and bisexual men might experience different challenges and lower health-related quality of life after prostate cancer treatment compared to heterosexual men4 (a provided ref.). It’s important to find healthcare providers who are knowledgeable and sensitive to these differences.*

[Copilot]

However, some of the care described resembled a structured summary, making it harder to distinguish differences from a compassionate perspective.

## Discussion

### Principal Findings

This study explored how 4 GenAI chatbots responded to a situated sexual health inquiry framed from the perspective of a gay man undergoing treatment for prostate cancer. Across platforms, chatbot responses varied in emotional attunement, degree of specificity, and attention to sexual minority–specific concerns, while consistently addressing treatment-related sexual issues and encouraging continued dialogue. Interactional features included inclusive language, provision of self-care advice, and explicit discussion of same-sex sexual practices. These variations were analytically organized using a 4-quadrant heuristic that maps emotional orientation and degree of specificity, providing an interpretive lens for understanding interactional diversity rather than for evaluating clinical performance or quality.

The findings indicate that when men who have sex with men seek health information following prostate cancer surgery, GenAI chatbots may differ not only in tone and communicative style but also in how interactional responses are organized and enacted. These differences were observed along the analytically derived dimensions of emotional attunement and informational specificity, shaping how guidance was framed and relationally positioned within the interaction. Such variation reflects different ways of presenting and contextualizing self-care–related information in sensitive health contexts, rather than stable or intrinsic properties of the systems themselves.

Building on existing research, studies on GenAI in health contexts have primarily focused on assessing the accuracy, safety, and technical performance of chatbot-generated information [9,10]. While these evaluations are essential, they offer limited insight into how health-related responses are framed and enacted in interaction, particularly in sensitive domains, such as sexual health. Consistent with emerging qualitative and nursing-oriented research on AI-mediated communication [11,12,24,25], the present findings suggest that GenAI chatbots may simulate supportive dialogue through tone, structure, and responsiveness, despite lacking ethical agency and clinical responsibility.

Importantly, the findings do not suggest that GenAI chatbots provide care in an ethical or caritative sense. Drawing on Nordic caritative caring theory [18,19] and prior analyses of caring encounters in technologically mediated contexts [20,26,27], the observed responses are better understood as care-like enactments. In this view, caring extends beyond the delivery of correct information and encompasses relational and contextual dimensions that may be partially simulated through language and interactional form. As Andtfolk [20] has noted, digital systems may perform aspects of care without consciousness, moral responsibility, or the capacity for genuine caring communion.

From a sociomaterial and actor–network perspective, these care-like enactments emerge from specific configurations of actors, practices, and technologies rather than residing within the chatbot itself [14-17]. Chatbot responses are shaped by prompts, platform design, training data, and user expectations rather than by stable or intrinsic properties of the system. Consequently, sexual health after prostate cancer treatment is not communicated as a single, stable phenomenon but is enacted differently depending on how concerns are articulated and responded to in particular human–technology interactions.

When considered alongside nursing theories of care, this sociomaterial understanding also aligns with self-care perspectives, such as Orem’s [28] theory, which emphasizes supporting individuals’ capacity to reflect on and manage health-related needs. In this study, GenAI chatbots did not provide care in an ethical or caritative sense [18,19], but their care-like enactments may be understood as digitally mediated supports for reflection, sense-making, and self-management within a broader care ecology. In this way, GenAI chatbots can be conceptualized as digital adjuncts that intersect with, but do not replace, professional care.

These considerations are particularly relevant for men who have sex with men following prostate cancer treatment. The findings highlight both the potential and the limitations of GenAI chatbots as digital adjuncts in this context. Sexual minority patients have documented unmet needs in sexual health, intimacy, and disclosure after treatment [1-5]. Several chatbot responses demonstrated sensitivity to same-sex practices and sexual minority–specific concerns, aligning with frameworks for LGBTQI+ (lesbian, gay, bisexual, transgender, queer, and intersex)-inclusive health care that emphasize recognition of sexual minority identities, practices, and relational contexts [29]. However, this sensitivity was inconsistently applied across platforms, underscoring that inclusivity in AI-mediated health communication is variable rather than assured by design.

The interactional patterns observed in chatbot responses can also be interpreted through the PLISSIT (Permission, Limited Information, Specific Suggestions, and Intensive Therapy) model, which outlines progressive levels of sexual health communication [30]. Across platforms, chatbot responses consistently operated within the first three levels of the model. They invited questions and normalized concerns about sexuality (Permission), provided general information about treatment-related sexual effects (Limited Information), and, in some cases, offered tailored guidance on same-sex practices and self-care strategies (Specific Suggestions). Notably, none of the chatbots reached the level of Intensive Therapy, which would require individualized clinical assessment, ethical responsibility, and professional accountability. This pattern aligns with interpreting chatbot responses as care-like enactments rather than clinical care. Furthermore, it supports conceptualizing GenAI chatbots as digital adjuncts that may facilitate dialogue and reflection on sexual health without substituting for professional nursing or therapeutic expertise.

### Strengths and Limitations

This study is based on a single, situated interaction per chatbot and does not aim to assess reproducibility, safety, or generalizability. The auto-netnographic approach involves interpretive judgment and reflexive engagement, which may introduce subjectivity [13,21]. Although no obviously fabricated claims were identified in this dataset, this absence should not be interpreted as evidence of accuracy, reliability, or safety, as prior studies have documented hallucinations and contextual errors in similar systems [9,25].

### Conclusions

The findings suggest that contemporary GenAI chatbots, when used as digital adjuncts, may enact communication styles perceived as supportive, culturally sensitive, and LGBTQI+-inclusive in specific sexual health interactions following prostate cancer treatment. Although these systems lack ethical consciousness and cannot replace professional care [24], their performative responses may complement clinical practice by facilitating reflection, dialogue, and access to sensitive information within a broader nursing and self-care context. Rather than demonstrating generalized performance or clinical reliability, this study highlights how care-like meanings may emerge in specific sociomaterial interactions between users and AI systems.

## Supplementary material

10.2196/81745 Multimedia Appendix 1 Prompts and initial answers on sexual health after prostate cancer treatment, specifically for men who have sex with men.

10.2196/81745 Multimedia Appendix 2 Prompts and specific answers on sexual health after prostate cancer treatment, specifically for men who have sex with men.

## Abbreviations

AI: artificial intelligence
CHART: Chatbot Health Advice Reporting Tool
GenAI: generative artificial intelligence
LGBT/LGBT+/LGBTQ/LGBTQI: lesbian, gay, bisexual, transgender, queer, and intersex
PLISSIT: Permission, Limited Information, Specific Suggestions, and Intensive Therapy
SRQR: Standards for Reporting Qualitative Research
