# Supplementary material for: Generative AI Chatbots as Digital Adjuncts for Sexual Health Information After Prostate Cancer in Men Who Have Sex With Men: Auto-Netnographic Study
Source: JMIR Cancer. 2026 Feb 9;12:e81745. doi: 10.2196/81745 (PMC12885184; doi:10.2196/81745)
Supplement: Multimedia Appendix 1 [file cancer-v12-e81745-s002.docx]

**Table 2: Prompts and specific answers on sexual health after prostate cancer treatment, specifically for men who have sex with men. Answers generated by the GenAI chatbots: ChatGPT, Claude, Copilot, and Gemini.**

|  | **ChatGPT** | **Claude** | **Copilot** | Gemini |
| --- | --- | --- | --- | --- |
|  | ChatGPT (GPT 4-technology), free of charge - GPT-4o. | Claude 3.5 Sonnet, free of charge | Microsoft Copilot GPT-4 Turbo | Google Gemini 2.0 Flash, free |
| *Are there any specific things I need to consider as a gay man regarding my sex life after treatment for prostate cancer?* | Yes, there are some unique considerations as a gay man when it comes to your sex life after prostate cancer treatment. While many issues are similar to those faced by heterosexual men, factors like sexual roles, intimacy dynamics, and community-specific expectations can play a role in your experience. Here are some key aspects to consider:  **1. Changes in Erectile Function and Penetrative Sex****  - If you’re the *insertive partner (top)*, erectile dysfunction (ED) could make penetration more difficult. Treatments like PDE5 inhibitors (Viagra, Cialis), injections, or penile implants may help.  - If you’re the *receptive partner (bottom)*, ED may be less of a concern, but decreased libido, loss of orgasm intensity, or changes in prostate sensation could impact pleasure.   **2. Prostate Sensation and Orgasm Changes**  - *Loss of prostate stimulation*: Many gay men incorporate prostate play into sex. After prostatectomy, the prostate is removed, so direct stimulation is no longer possible. However, some men still enjoy anal play by stimulating surrounding areas like the perineum or rectal nerves.  - *Orgasms feel different:* Even if you can still climax, orgasms may feel less intense or "dry" due to the absence of semen. Some men describe orgasms as shorter or less pleasurable, though this varies.   **3. Anal Sex and Bowel Function**  - *After surgery or radiation, some men experience changes in bowel control or increased sensitivity in the rectal area.* If you engage in receptive anal sex, you may need to monitor how your body reacts and adjust as necessary.  - *Radiation can cause rectal irritation, thinning, or scarring*, making anal penetration less comfortable. Using plenty of lubricant and allowing time for healing is essential.   **4. Testosterone & Libido Concerns**  - Hormonal therapy (ADT) lowers testosterone, which may reduce your sex drive and energy levels. This can impact both your desire for sex and your ability to engage in it confidently.  - Some doctors may eventually consider *testosterone replacement therapy (TRT)* if your cancer is well-controlled, though this requires careful monitoring.   **5. Emotional and Psychological Aspects**  - *Body image changes*: Weight gain, muscle loss, or fatigue from hormonal therapy can affect self-esteem, particularly in gay spaces where youth and fitness are often emphasized. Staying active and focusing on self-acceptance can help.  - *Changes in sexual identity and confidence*: If you strongly associate your sexual identity with a specific role (e.g., top or bottom), treatment side effects might challenge how you see yourself sexually. Exploring new ways of pleasure can help redefine your sex life.   **6. Community and Dating Considerations**  - *Dating and disclosure*: If you're single, you might wonder how and when to share your medical history with new partners. Some men fear rejection, but others find that honesty fosters deeper connections.  - *Navigating hookup culture*: If you engage in casual sex, you may need to communicate openly about changes in function and pleasure. Some men explore alternative activities, like mutual masturbation or non-penetrative sex.   **7. Support and Resources**  - Finding support groups for gay men with prostate cancer can help you navigate these challenges with others who understand. Organizations like *Malecare* offer LGBTQ+-friendly cancer support.  - *Sex therapy or counseling* with someone knowledgeable about LGBTQ+ sexuality can be beneficial in working through both physical and emotional changes.   **Final Thoughts**  Your sex life will likely change, but that doesn’t mean it has to end. Many men discover new ways to experience pleasure, whether through adapting physically, redefining intimacy, or exploring different forms of touch and connection. If you’d like, I can help you find LGBTQ+-friendly doctors, sex therapists, or resources specific to your area. | Yes, there are several important considerations specific to gay men dealing with prostate cancer treatment:  Anal Sex: - If you engage in receptive anal sex, this can generally be resumed after healing from either surgery or radiation (usually 4-6 weeks for surgery, timing varies for radiation) - If you engage in insertive anal sex, erectile challenges may affect this more significantly than some other forms of sexual activity - Some men find they need to adjust their preferred sexual roles based on treatment effects  Oral Sex: - Can generally be resumed after healing - Changes in orgasm sensation might affect how you enjoy this - No ejaculate after surgery means different sensations and cleanup  Other Considerations: - Gay men may feel particular pressure around maintaining erectile function due to cultural expectations - Dating or meeting new partners may feel more challenging if you're experiencing sexual side effects - The gay community can sometimes place high value on sexual performance, which might add stress during recovery  Communications with Partners: - Being open about changes in sexual function is important - Many men find their partners are understanding and willing to explore new forms of intimacy - If you're in the dating scene, you might want to think about how and when to discuss these changes  Support: - Standard prostate cancer support groups might focus heavily on heterosexual experiences - Looking for LGBTQ+-specific cancer support can provide more relevant perspectives - Some cities have specific support groups for gay men with prostate cancer  Would you like to discuss any of these aspects in more detail? Also, are there particular sexual activities or roles that are especially important to you that you're concerned about maintaining? | Yes, there are some specific considerations for gay men regarding their sex life after prostate cancer treatment. Here are a few key points:  1. Communication with Healthcare Providers Find LGBTQ+-Friendly Providers: It's important to work with healthcare providers who are knowledgeable about the unique concerns of gay men. This can help ensure that your specific needs and questions are addressed12. Discuss Sexual Orientation: Make sure your healthcare provider is aware of your sexual orientation and any specific sexual practices. This can help them provide more tailored advice and care1. 2. Impact on Sexual Practices Anal Intercourse: If you engage in receptive anal intercourse, radiation therapy might affect the rectal area, potentially causing discomfort or other issues. Discuss these concerns with your doctor to understand the risks and how to manage them2. Prostate Stimulation: If you enjoy prostate stimulation, be aware that treatments like surgery or radiation can affect the prostate's sensitivity and function2. 3. Psychological and Emotional Support Mental Health: The psychological impact of prostate cancer and its treatment can be significant. Consider seeking support from a therapist who understands the experiences of gay men1. Support Groups: Joining support groups specifically for gay men with prostate cancer can provide a sense of community and shared understanding2. 4. Managing Side Effects Erectile Dysfunction (ED): Both surgery and radiation can lead to ED. Medications, penile rehabilitation, and other treatments can help manage this1. Libido and Hormone Therapy: Hormone therapy can reduce libido. Discuss potential treatments or strategies to manage this with your healthcare provider1. 5. Open Communication with Partners Discuss Changes: Openly discuss any changes in your sexual function or desires with your partner. This can help maintain intimacy and understanding in your relationship2. Explore Alternatives: Be open to exploring different forms of sexual expression and intimacy that might be more comfortable or satisfying post-treatment1. Resources Prostate Cancer UK: They offer specific information and support for gay and bisexual men2. Facing Our Risk of Cancer Empowered (FORCE): Provides resources and support for LGBTQ+ individuals with cancer1. Navigating these changes can be challenging, but with the right support and information, you can maintain a fulfilling sex life. If you have more questions or need further assistance, feel free to ask. | It's important to acknowledge that while the core medical aspects of prostate cancer treatment are the same for all men, there are specific considerations for gay men regarding their sexual health. Here's a breakdown of those key points:     Key Considerations for Gay Men:  Impact on Receptive Anal Intercourse: The prostate plays a significant role in the pleasure experienced during receptive anal intercourse. Therefore, any treatment that affects the prostate can have a notable impact on this aspect of sexual activity.    Changes in sensation, discomfort, or pain may occur. If radiation therapy is used, especially brachytherapy (seed implants), there are specific concerns about potential radiation exposure to a partner during anal intercourse, requiring careful adherence to medical advice regarding safe timing and practices.    Changes in Sexual Roles: Some research indicates that prostate cancer treatment can lead to changes in sexual roles among gay men. For example, some men may shift from being the insertive partner to the receptive partner, or vice versa, due to changes in sexual function.    These changes can have emotional and psychological implications, and it's essential to address them openly with your partner and healthcare providers. Emotional and Psychological Impact: The potential for changes in sexual function can have a significant emotional impact, potentially affecting self-esteem and body image. Gay men may experience unique concerns related to masculinity and sexual identity.    It is very important to seek out support groups, or mental health professionals that are familiar with LGBTQ+ needs. Communication with Healthcare Providers: Open and honest communication with your healthcare team is crucial. It's essential to find providers who are knowledgeable and sensitive to the specific sexual health needs of gay men. Many men find that their doctors do not ask about their sexual orientation, so it is important for you to be proactive in those conversations. Lack of Targeted Information: Historically, much of the information and support materials related to prostate cancer have been geared toward heterosexual men. This can lead to feelings of isolation and a lack of access to relevant resources. It is important to seek out LGBTQ+ focused resources. Recommendations:  Seek out LGBTQ+ affirming healthcare providers: Look for urologists and oncologists with experience in treating gay men. Be open and honest with your medical team: Discuss your sexual activity and concerns openly. Find LGBTQ+ specific support resources: Organizations like the National LGBT Cancer Network offer valuable information and support.    Consider pelvic floor therapy: This therapy can help with post operative continence, and can also help with some sexual function. By addressing these specific considerations, you can take proactive steps to maintain your sexual health and quality of life after prostate cancer treatment. |
